# Using Landsat satellite data to support pesticide exposure assessment in California

**DOI:** 10.1186/1476-072X-9-46

**Published:** 2010-09-16

**Authors:** Susan K Maxwell, Matthew Airola, John R Nuckols

**Affiliations:** 1 BioMedware, Inc., Ann Arbor, Michigan, USA; 2 Occupational and Environmental Epidemiology Branch, Division of Cancer Epidemiology and Genetics, National Cancer Institute, National Institutes of Health, Department of Health and Human Services, Bethesda, Maryland, USA; 3 Westat, Inc., Rockville, Maryland, USA; 4 Department of Environmental and Radiological Health Sciences, Colorado State University, Fort Collins, Colorado, USA

## Abstract

**Background:**

The recent U.S. Geological Survey policy offering Landsat satellite data at no cost provides researchers new opportunities to explore relationships between environment and health. The purpose of this study was to examine the potential for using Landsat satellite data to support pesticide exposure assessment in California.

**Methods and Results:**

We collected a dense time series of 24 Landsat 5 and 7 images spanning the year 2000 for an agricultural region in Fresno County. We intersected the Landsat time series with the California Department of Water Resources (CDWR) land use map and selected field samples to define the phenological characteristics of 17 major crop types or crop groups. We found the frequent overpass of Landsat enabled detection of crop field conditions (e.g., bare soil, vegetated) over most of the year. However, images were limited during the winter months due to cloud cover. Many samples designated as single-cropped in the CDWR map had phenological patterns that represented multi-cropped or non-cropped fields, indicating they may have been misclassified.

**Conclusions:**

We found the combination of Landsat 5 and 7 image data would clearly benefit pesticide exposure assessment in this region by 1) providing information on crop field conditions at or near the time when pesticides are applied, and 2) providing information for validating the CDWR map. The Landsat image time-series was useful for identifying idle, single-, and multi-cropped fields. Landsat data will be limited during the winter months due to cloud cover, and for years prior to the Landsat 7 launch (1999) when only one satellite was operational at any given time. We suggest additional research to determine the feasibility of integrating CDWR land use maps and Landsat data to derive crop maps in locations and time periods where maps are not available, which will allow for substantial improvements to chemical exposure estimation.

## Background

A primary emphasis of our research is to examine the relationship between pesticides used on crops grown near individual residences and health outcomes in California. Increased detection and/or concentration of pesticides used on crop fields adjacent to residences in biological samples of residents and house dust have been shown in several studies [1-4]. Methods for estimating pesticide exposure are needed since environmental samples of pesticide levels in the home can only be collected after enrollment into a study (i.e., after date of diagnosis). Determining the transport and fate of pesticide chemicals is a complex process which depends on many factors such as weather conditions, vegetation characteristics, soil properties, application method, and chemical persistence [5]. The condition of the land cover (e.g., bare soil, vegetation) at the time of chemical application has been shown to have a significant effect on chemical drift [6]. Land use and land cover maps (e.g., urban, crop type), such as provided by the California Department of Water Resources (CDWR), have been used in geographic-based transport and fate models to derive estimates of human exposure [3,4,7]. However, land cover data at the frequency and resolution necessary for exposure assessment are often not available during the critical pre-diagnosis exposure periods of most epidemiological studies (e.g., date of conception until date of diagnosis in birth outcome studies).

There are many satellite sensor systems that collect earth surface measurements at varying spatial resolutions and temporal frequencies which can be used to determine land cover conditions [8]. Sensor systems such as the Advanced Very High Resolution Radiometer (AVHRR) and Moderate Resolution Imaging Spectroradiometer (MODIS) instruments are commonly used to study land cover phenology because of their daily repeat coverage [9]. However, the low spatial resolution of these sensors (250 m for MODIS, 1 km for AVHRR) severely limits the capability of measuring individual crop field properties. Landsat images have a relatively high spatial resolution (60 m for Landsat 1, 2, and 3; 30 m for Landsat 4, 5, and 7) enabling land cover and land use characterization at the local scale [10]. In addition, Landsat data have been collected since 1972 which allows for reconstructing historical exposures to study health effects that have a long latency period, such as many cancers.

Landsat data are collected every 8 or 16 days, depending on the number of sensors in orbit, providing relatively frequent measures of land cover conditions. Prior to 1999 only one sensor was in orbit at any one time providing only 16-day repeat coverage. Since 1999, two Landsat sensors have been in orbit (Landsat 5 and 7) providing 8-day repeat coverage. Although an instrument failure on the Landsat 7 sensor on May 31, 2003 resulted in a data loss of approximately 22% of the scene area, effective gap-fill processes are available to interpolate the missing data [11]. One of the limitations of using multi-temporal Landsat data in the past has been the cost of purchasing the images [10,12]. The US Geological Survey (USGS) recently made Landsat free of charge [13] eliminating one of the major barriers to using the data for studies requiring frequent measures of land cover conditions.

The purpose of this study was to explore the potential for using Landsat satellite data to support pesticide exposure assessment in California. This paper presents the results of our study of 17 major crop types and provides an extended discussion on the strengths and limitations of the CDWR land use maps and Landsat image data for pesticide exposure assessment in California.

## Methods

### Study area description

**Figure 1:**
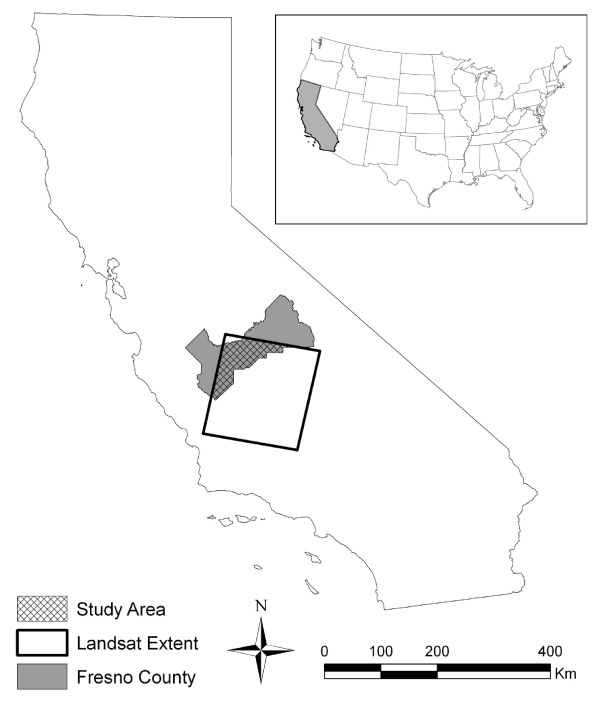
**Study area (intersection of Fresno County and Landsat image)**.

Our study area covered a 3,346 km^2 ^agricultural region within Fresno County, located in the southern region of the Central Valley California (Figure 1). Factors such as climate, soil conditions, cultivation, and irrigation practices in this area allow for a wide variety of crop types, where double- and triple-crops can be grown on a field within a single year [14,15]. 97.4% of crops grown in 2002 in Fresno County were irrigated [16]. Major crops in the western portion of the study area were primarily field crops (e.g., cotton, corn, and sugar beets), vegetable crops (e.g., tomatoes, cantaloupe), grain (e.g., wheat, barley, oats) and alfalfa. Vineyards and citrus, fruit, and nut orchards were the dominant crops grown in the eastern region of the study area. Climate conditions were normal for the year 2000 (the year of our study) except during January and February when abnormally dry climate conditions were reported (http://drought.unl.edu/dm/archive.html; accessed May 12, 2010).

### Landsat image selection and pre-processing

Landsat 5 and 7 images for the year 2000 were selected and downloaded from the U.S. Geological Survey (USGS) using the Global Visualization Viewer (GloVis) website (http://glovis.usgs.gov/; accessed May 12, 2010). Of the 44 Landsat images available in the year 2000, 24 were selected because they had low- or no-cloud cover over our study area in the northwest portion of the scene (Table 1). The majority of images excluded were during the winter months where 11 of the 14 images available during the November through February time period contained substantial cloud cover in our study area.

**Table 1:** Summary of Landsat satellite image characteristics for the year 2000 used in the study (Path/Row 042/035)

Image #	Landsat Sensor	Landsat Acquisition Date	Day of Year	% Cloud Cover	Crops excluded due to cloud cover
1	7	Feb 2	33	0	
2	7	Mar 21	81	0	
3	5	Mar 29	89	10	Oranges
4	7	Apr 6	97	3	
5	7	Apr22	113	10	All orchard crops except pistachios, vineyards, idle, mixed pasture
6	5	Apr30	121	0	
7	5	Jun 1	153	0	
8	5	Jun 17	169	0	
9	7	Jun 25	177	0	
10	5	Jul 3	185	0	
11	7	Jul 11	193	0	
12	5	Jul 19	201	0	
13	7	Jul 27	209	0	
14	5	Aug 4	217	0	
15	7	Aug 12	225	0	
16	5	Aug 20	233	0	
17	7	Aug 28	241	17	
18	5	Sep 21	265	0	
19	7	Sep 29	273	0	
20	5	Oct 7	281	0	
21	7	Oct 15	289	57	Onions/garlic, mixed pasture, oranges, peaches/nectarines, plums
22	5	Oct 23	297	0	
23	7	Nov 16	321	1	
24	5	Dec 10	345	30	Cotton, sugar beet, onions/garlic, tomato, grain/hay, oranges

The images were reprojected to Albers Conical Equal Area using cubic convolution resampling at 30 meter spatial resolution and then radiometrically corrected to at-sensor reflectance to correct for seasonal reflectance variances caused by sun angle and distance [17] using ERDAS Imagine version 9.3 software. The Normalized Difference Vegetation Index (NDVI) derived from the red visible Band 3 and near infrared Band 4 ((B4-B3)/(B4+B3)) was used to characterize crop phenology [18]. The NDVI is one of the most widely used vegetation indices to monitor seasonal changes in vegetation growth [19]. The NDVI represents a measure of canopy 'greenness' where values below 0.1 are generally non-vegetated surfaces such as bare soil or snow and dense green vegetation canopies are generally greater than 0.6 [20]. Clouds, cloud shadows, and haze within each image were eliminated by visually inspecting the NDVI image alongside a multi-spectral image, delineating the affected regions, and recoding the pixels within the region to a value of 255. The NDVI images were then stacked into a single 24-layer file and an intersect function applied to retain only those areas where image data was available for all time periods.

### California Department of Water Resources (CDWR) land use map

A crop map for the year 2000 for Fresno County, generated by the CDWR, was used as our ground reference map (http://www.water.ca.gov/; accessed May 12, 2010). CDWR maps are produced for counties with high agricultural land use about every 7-10 years (http://www.water.ca.gov/landwateruse/lwudatacoll.cfm; accessed May 12, 2010). CDWR personnel delineated boundaries of the same land use type using aerial photographs collected in mid June 2000. CDWR field crews visited nearly all polygons at least once during the period July through October 2000 and recorded the land use within each polygon as a specific crop type, crop type group, or land cover type. The minimum mapping unit of the map was 0.81 hectares.

### Sample selection method

We selected 17 of the 64 crop types listed in the CDWR map. These 17 crop types constitute 90.1% of the total area of cropped land in Fresno County. A stratified random sample approach was used to select 30 sample polygons for each of the 17 crop types. Only polygons that were designated as single cropped, greater than 60 meters in width, at least 4.0 hectares in area, and labeled as a specific crop type (e.g., 'cotton' or 'vineyard') were included in the sample selection process. Polygons labeled with a general crop class, such as 'field crop', or labeled as double-cropped (n = 18), inter-cropped (n = 32), or mixed land use (n = 21) were excluded. Only crops with at least 30 polygons meeting the above criteria were selected for evaluation in our study (Table 2). Thirty polygons were randomly selected for each of the 17 crops. One pixel (900 m^2^) per polygon was selected at the location of the polygon label point except in the case where the spectral tone of the pixel was not representative of the field (e.g., field edge, field access road). We ensured that a representative pixel was selected by overlaying the CDWR label point for each CDWR polygon over the satellite image and visually inspecting the sample location. Only one pixel was selected, as opposed to multiple pixels, to limit NDVI measurements that represented mixed land cover types at any one point in time over the year, such as variable harvest dates or mid-year crop field boundary changes.

**Table 2:** List of CDWR crop types selected within study area

Crop #	Crop Group/Crop Name	CDWR Code	# CDWR polygons	Median polygon size (h)	Total Hectares	% of Total Hectares
	Field crop					
1	Cotton	F1	720	34.5	63,914	20.5
2	Sugar beet	F5	80	50.5	4,060	1.3
3	Corn	F6	415	13.3	4,465	1.4
						
	Truck, Nursery, Berry Crop					
4	Melons, squash, cucumbers	T9	110	10.8	3,150	1.0
5	Onions, garlic	T10	156	45.1	7,456	2.4
6	Tomato	T15	259	60.7	19,073	6.1
						
7	Idle	I1	251	4.3	2,111	0.7
						
8	Grain and Hay	G0	277	32.0	18,398	5.9
						
	Pasture					
9	Alfalfa and alfalfa mixtures	P1	620	18.8	22,302	7.1
10	Mixed Pasture	P3	408	5.3	3,571	1.1
						
	Citrus and Subtropical					
11	Oranges	C3	1,912	5.0	13,135	4.2
						
	Deciduous Fruits and Nuts					
12	Peaches, nectarines	D5	2,231	3.9	15,808	5.1
13	Plums	D7	1,544	3.4	7,683	2.5
14	Almonds	D12	1,051	9.8	22,429	7.2
15	Walnuts	D13	212	6.6	2,137	0.7
16	Pistachios	D14	101	8.3	3,010	1.0
						
17	Vineyards	V0	6,006	6.9	99,772	31.9
						
	Total				312,475	

We generated a time series of NDVI values for each of the sample locations by intersecting the *x, y *location of the polygon label point with the 24-layer stack of NDVI images. We evaluated the phenological properties of the field samples using time series plots and summarized the properties for each crop class using box plots of the median, 25^th ^(Q1) and 75^th ^(Q3) quartiles, minimum, and maximum NDVI values. We excluded crops in some time periods because an insufficient number of samples (< 10 polygons) were collected due to cloud cover (Table 2).

## Results

### Field crops (cotton, sugar beets, corn)

**Figure 2:**
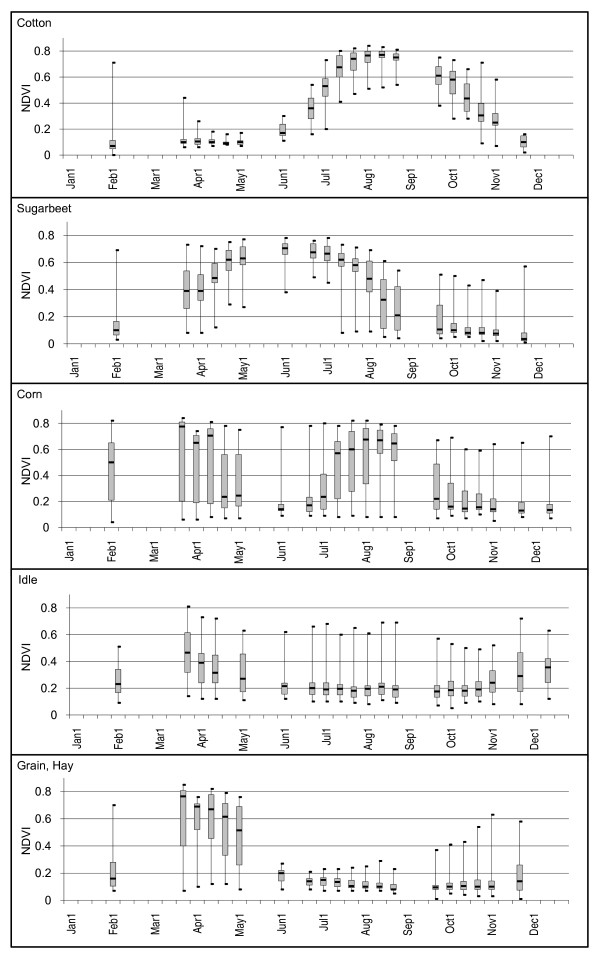
**NDVI time-series box plots for selected crops**. Plots are shown for fields classified as cotton, sugar beet, corn, idle, and grain/hay in the 2000 CDWR land use map for Fresno County, California. Minimum, maximum, median, 1^st ^and 3^rd ^quartiles are noted.

The dominant crop in our study area was cotton (20.5% of total area of CDWR polygons selected). NDVI values for cotton field samples had the lowest variability for all time periods (median Q3-Q1 = 0.09) of all the crops we evaluated. NDVI values for the samples identified as cotton by CDWR were very low (< 0.20) from early February until the first of June indicating bare soil. NDVI values then increased sharply from June to July, peaking in early August (median NDVI = 0.77), and returning to NDVI values representative of bare soil by late November (Figure 2).

NDVI values for sugar beet and corn samples peaked earlier in the season as compared to cotton (early June and late March respectively) and were more highly variable during periods of green-up and harvest, or senescence. The sugar beet samples were most variable from late March to early April (Q3-Q1 NDVI values of 0.28 and 0.19 respectively) and early August through late September (median Q3-Q1 NDVI = 0.28).

We found several samples, particularly in the corn crop (n = 24) that exhibited NDVI time series depicting two or more green-up periods (Table 3) even though we had eliminated CDWR polygons from our study designated as multi-cropped. Samples were identified as multi-cropped if NDVI values increased then decreased (or vice versa) more than once over the year. Two growth/harvest periods were depicted in the NDVI time series for 23 of the 30 field samples. An early season crop cycle period occurred during the first half of the year with harvest typically completed by early June. Times of vegetation green-up and crop harvest were highly variable for the second crop cycle, yet fields were generally at peak greenness by early August and harvested by mid-October. Vegetation conditions were highly variable between fields during most of the year (Q3-Q1 > 0.40 for 10 of the 24 time periods). Only five of the 30 CDWR samples classified as corn were single-cropped with peak growing times typically occurring during late June. The NDVI time-series for one sample classified as corn showed no indication of green vegetation over the entire year (all NDVI values < = 0.20).

**Table 3:** Number of crops grown within the field indicated by periodic high/low NDVI values across the year

Crop #	Crop Group/Crop Name	One	Two	Three	No crop
	Field crop				
1	Cotton	29	1		
2	Sugar beet	29	1		
3	Corn	5	23	1	1
					
	Truck, Nursery, Berry Crop				
4	Melons, squash, cucumbers	18	12		
5	Onions, garlic	26	3		1
6	Tomato	28	2		
					
7	Idle	18	9		3
					
8	Grain and Hay	19	4		7

### Idle land

Of the 30 field samples classified as idle in the CDWR map, only three indicated NDVI values representative of bare soil over the entire year (< 0.31). The remaining 27 fields contained periods where NDVI values indicated green vegetation at least once during the year (maximum NDVI values ranged from 0.39 to 0.81). The first green-up period was early in the year (beginning early February and ending late May) and a second period occurred late in the year (beginning late October and ending early December or later).

### Grain/hay

Most grain/hay (e.g., wheat, barley, oats, mixed grain and hay) field samples (n = 22) depicted an early season growth cycle, beginning in early February (median NDVI = 0.23), peaking in late March (median NDVI = 0.80), and completing by late June (median NDVI = 0.17). Four of the 30 field samples showed the early season crop cycle was followed by another green-up cycle beginning in early September (NDVI < 0.20 followed by NDVI > 0.45) indicating a second crop was grown. Of the 277 polygons labeled as grain/hay in the CDWR map, 13 were reported as double-cropped by CDWR, with the second crop identified as corn (n = 6), sudan (n = 6), or melons/squash/cucumbers (n = 1). Seven samples never exceeded NDVI values over 0.35 for the entire year suggesting that no crop was grown in these fields. Eliminating these fields reduced variability in NDVI samples considerably for the March through April time period (from maximum difference of 0.41 to 0.15).

### Truck crops (melons/squash/cucumbers, onions/garlic, tomato)

**Figure 3:**
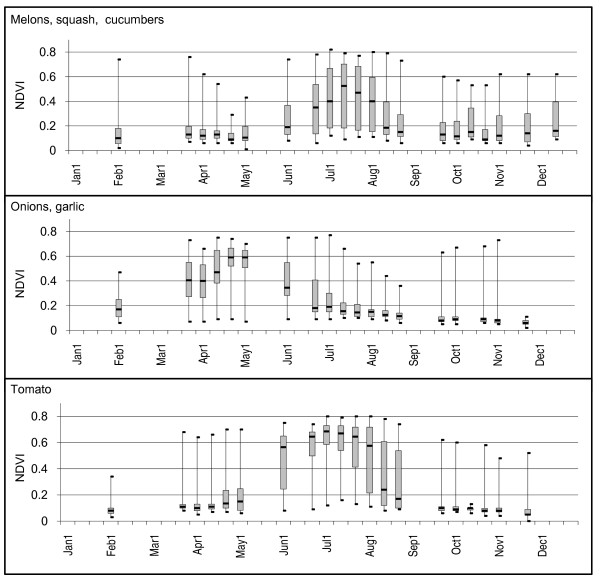
**NDVI time-series box plots for selected crops**. Plots are shown for fields classified as melons/squash/cucumbers, onions/garlic, and tomato in the 2000 CDWR land use map for Fresno County, California. Minimum, maximum, median, 1^st ^and 3^rd ^quartiles are noted.

NDVI time series for the fields classified as onions/garlic or tomatoes primarily depicted single cropped fields (n = 26 and n = 28 respectively) (Figure 3). Peak greenness generally occurred in late April to early May for onions/garlic samples and early July for tomato samples. Both classes showed high variability in NDVI values during the time period of vegetation growth and harvest. The growing season for the melons/squash/cucumber crop group occurred typically between mid-May and mid-August yet NDVI values were highly variable (Q3-Q1 differences of 0.40 or greater for five of the six time periods). Twelve of the 30 field samples had NDVI patterns depicting two crop growing cycles, with approximately half occurring early in the year (February through April) and half late in the year (late September through mid-December).

### Orchards and Vineyards

**Figure 4:**
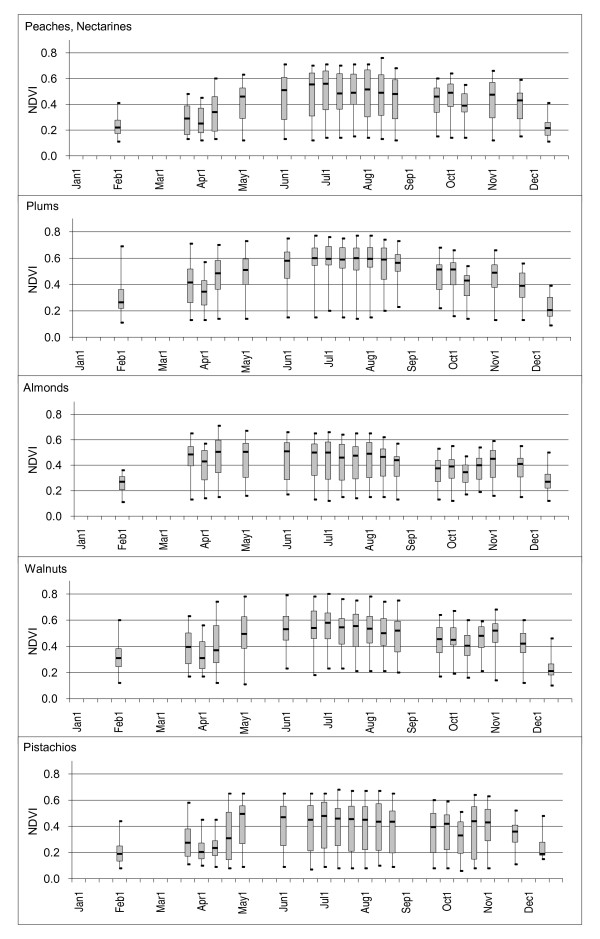
**NDVI time-series box plots for selected crops**. Plots are shown for fields classified as orchards in the 2000 CDWR land use map for Fresno County, California. Minimum, maximum, median, 1^st ^and 3^rd ^quartiles are noted.

**Figure 5:**
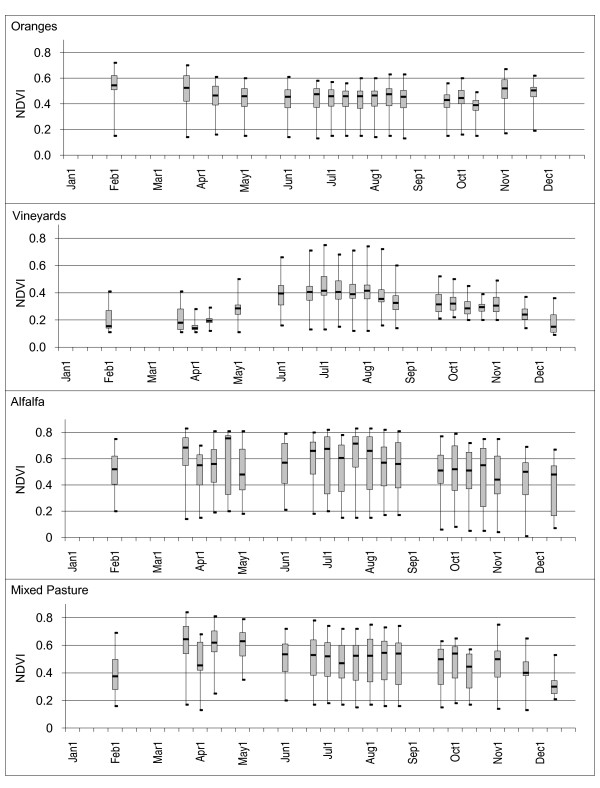
**NDVI time-series box plots for selected crops**. Plots are shown for fields classified as oranges, vineyards, alfalfa, and mixed pasture in the 2000 CDWR land use map for Fresno County, California. Minimum, maximum, median, 1^st ^and 3^rd ^quartiles are noted.

**Figure 6:**
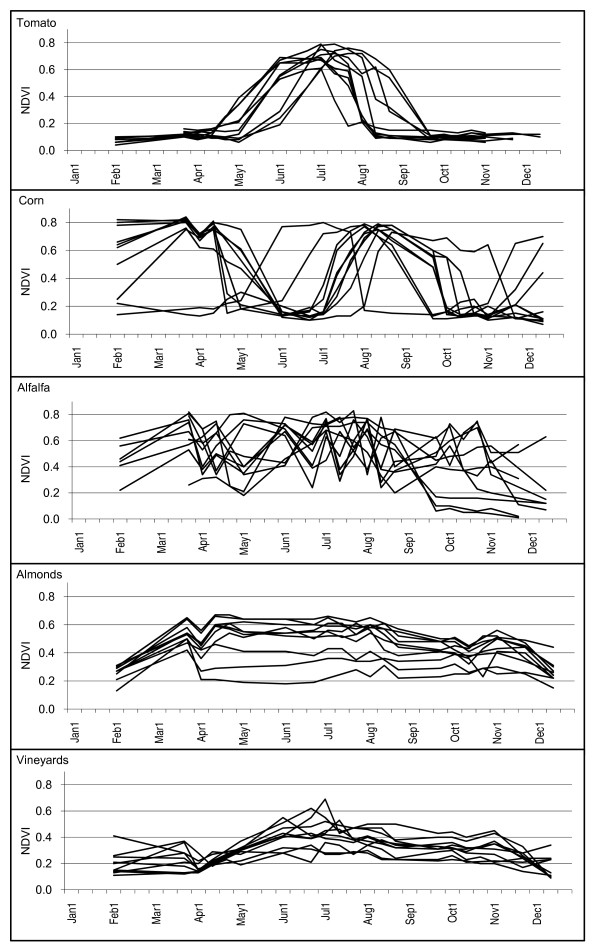
**NDVI time-series plots for selected crops**. Fields classified as tomatoes, corn, almonds, alfalfa, and idle in the 2000 CDWR land use map for Fresno County, California are shown. Values were interpolated between time periods for visual clarity.

Orchard crop samples demonstrated relatively long and flat NDVI patterns from May through November where median NDVI values ranged from 0.33 to 0.60. Each field sample demonstrated slow increasing and decreasing trends in NDVI values over the early and late periods of the year where values rarely dropped below 0.20 (Figures 4, 5, and 6). NDVI values were highly variable over most of the year for most of the orchard crops, particularly with pistachios (median NDVI Q3-Q1 difference = 0.29) possibly due to the distribution in ages of the fields. Oranges and vineyards had relatively low within class variability as compared to the other orchard crops (median NDVI Q3-Q1 differences = 0.12 and 0.10 respectively).

### Alfalfa and Pasture

NDVI values were highly variable throughout the year for alfalfa fields resulting from multiple harvest/re-growth cycles that are typical of this crop. Alfalfa can be harvested up to eight times in California [14]. NDVI values of 0.70 followed by NDVI values of 0.40 in the next time period were common in alfalfa fields. High variability in NDVI values across the entire growing season were also shown in the mixed pasture class. In this class, the variability was due to the relative difference between the NDVI time-series of individual fields, similar to the orchard and vineyard classes, as opposed to variability resulting from vegetation harvest/re-growth cycles, such as in the alfalfa class (Figure 6).

## Discussion

### Using Landsat data to identify ground cover condition at the time of pesticide application

**Figure 7:**
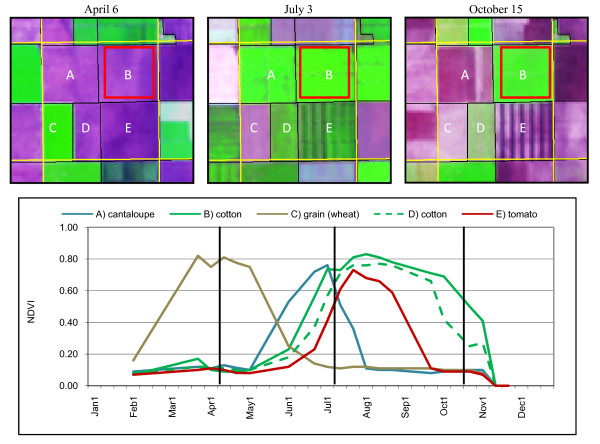
**Landsat images depicting crop field conditions at or near the time of pesticide applications to a cotton field (B, outlined in red)**. Dates of images from left to right are April 6, July 3, and October 15, 2000. Purple tones indicate bare soil and green tones indicate green vegetation. Crop fields were labeled in the CDWR map as A) cantaloupe, B) cotton, C) grain (wheat), D) cotton, E) tomato. Black lines in the image are the CDWR land use boundaries and yellow lines are Section boundaries. Bottom graph shows NDVI time-series plots for the crops. NDVI values were interpolated for missing time periods for visual clarity. The Landsat image dates depicted in the three images at top are shown as black vertical lines on the graph.

The California Pesticide Use Reporting (PUR) database is the primary source of information on where, when, and how pesticides are used in California [21]. Each PUR record contains information on the type of chemical applied, the type of crop the chemical was applied to (e.g., cotton, tomato) and the number of acres planted, among other attributes. Identifying crop field conditions at the time of pesticide application could potentially improve predictions of chemical movement in the environment. For example, chemicals sprayed on a crop field of small plants in the early growth stage, may have different drift patterns as compared to a field where plants form a dense vegetation canopy. Figure 7 shows Landsat images on, or near, three different dates when pesticides were applied according to PUR data records. The Landsat images are displayed using a three-band color combination (red using the middle-infrared band 5, green using the near-infrared band 4, and blue using the red visible band 3) where green tones represent green vegetation and purple tones represent bare soil. Bright green tones (e.g., July 3 image, crop fields labeled A, B, and D) represent dense green crop canopy whereas lighter green tones (e.g., Field D in the October 15 image) represent crops with only partial green canopy cover (i.e., contain a mixture of bare soil and green vegetation).

Pesticide use records noted that PCNB (pentachloronitrobenzene), a soil fungicide, was applied on April 2 to the cotton field labeled as Field B [21]. Notice that the cotton field and most of the surrounding crop fields were bare soil. On July 5 mepiquat chloride, a plant growth regulator, was applied to the same cotton field (B) when the crop was at full canopy cover and the surrounding fields are at various stages of vegetation, from bare soil to full green canopy (Figure 7, middle image). A third date, October 15, is represented in the image on the right when a pesticide mixture (cyclanilide, ethephon, etc.) was applied. Similar to the April 6 image, most of the surrounding fields were bare soil.

Although we were able to determine crop field conditions over most of the year, clouds prohibited complete coverage over the winter months. We reviewed Landsat 5 image cloud statistics for all years from 1990 through 2008 and found that approximately 60 percent of scenes had more than 30 percent cloud cover for the months December and January. A recent review by Ju and Roy (2008) evaluating the characteristics of clouds in Landsat 7 imagery supports this finding [22]. Characterizing crop field conditions prior to 1999, especially during winter months, will be limited because only one Landsat satellite will be operational at any given time. The AVHRR and MODIS sensors provide daily repeat coverage, yet the spatial resolution of this imagery (1 km and 250 m respectively) is likely too course to measure individual field characteristics in this region (Table 1). One AVHRR pixel would depict only a single measure for all five crop fields depicted in Figure 7.

### Using Landsat data to determine multi-cropped fields

Climatic conditions, irrigation practices, and soil conditions in Fresno County allow for the possibility of more than one crop to be grown on a field within a single year [14,15]. Exposure models that do not consider pesticides applied to all crops grown over the year may underestimate modeled chemical exposure. The CDWR crop maps were developed by visiting the field only once during the year, typically between July and October, and aerial photographs were used to identify crops grown on fields at other times of the year [23]. We found that the phenological patterns of many samples classified as corn, melons/squash/cucumbers, idle, and grain/hay in the CDWR map had two distinct green cycles. The majority of CDWR samples classified as corn (76.7%) indicated two crops were grown in the same field over the year and a substantial number of melon/squash/cucumber (40.0%) and idle (30.0%) samples also exhibited double-cropped phenological patterns. USDA Weekly Weather and Crop Reports for California note that fields planted as wheat early in the year were followed by second crops of dry beans, corn, or wheat (for May 9, May 23, June 27, and July 4 [14]), supporting our findings, although increases in NDVI values could also be a result of weed growth within the field. Several samples classified as grain/hay (23.2%) showed no distinct green-up period over the year suggesting these fields may have been incorrectly classified in the CDWR. Integrating Landsat time-series data with the CDWR map would enable the identification and flagging of potential misclassifications.

### Using Landsat data for crop type identification

Pesticide exposure assessment studies are typically done in retrospect [3,7,24,25] where historical crop maps are required. The ability to identify specific crop types grown near individual residences is a key element for improving the geographic scale of modeled pesticide exposure in California [26-28]. While the State of California maintains one of the most comprehensive pesticide use reporting databases in the world [21], the data is only recorded at a geographic scale of an approximately 2.6 km^2 ^polygon, or "Section" in the U.S. Public Land Survey, which may not be optimal for exposure assessment [4,28,29]. The CDWR crop maps have been shown to be useful for identifying the location of pesticide applications [28,29] and improving the predictions of pesticide detection and concentration in residential carpet dust [4], however these maps are only available at the county-level for intermittent years, and as we found in our study, did not adequately identify idle or multi-cropped fields. For Fresno County, CDWR maps are only available for three years from 1986 to present (1986, 1994, and 2000). Crops planted can change from year-to-year and therefore crop maps for every year are needed to accurately identify the location of pesticide use.

The wide variety of crops grown in California, where double- and triple-plantings are possible, makes this a challenging region to identify individual crop types. For our study area, the 2000 CDWR map for Fresno County listed 64 agricultural crop classes and many of those were crop groups or generic classes, such as miscellaneous field crops. Landsat image data has a long history of being useful for crop type identification in the U.S. [12] although very few studies were found in California. The only published study we found in the literature was by Congalton et al (1998) who used Landsat 5 data for mapping 13 crop classes along the Colorado River in southeastern California [30]. Crops were mapped three to four times per year from 1994 to 1997 to support a consumptive water use application. A supervised classification approach was used which was supported by extensive ground reference data collected at, or near, the Landsat overpass date. Overall accuracy for 12 dates ranged from 93.0% to 95.0% with individual class accuracies varying widely depending on the date used. The high spectral variability in orchard crops required the manual interpretation of aerial photographs. Lettuce and crucifers (broccoli, cauliflower, cabbage, and bok-choy) were classified using images only collected during the winter months.

A crop map for the entire state of California was recently produced by the USDA using a time series of 2007 Landsat data [31]. Several Landsat dates from mid-April to early September (using bands 1-5, 7 in each date) were used in the classification process. MODIS image data collected over the winter months was used to improve the identification of winter wheat. Overall kappa accuracy of 0.967 was reported for all land cover classes including 64 crop types, although this may be over estimated as the training and validation was not performed independently (Patrick Willis, USDA, personal communication). A wide variety of orchard crops, rare crops, and two double-cropped classes (winter wheat/corn, oats/corn) were classified. All of the crops such as onions, garlic, watermelon, and cantaloupe were classified individually as opposed to being lumped into a crop group (e.g., onions/garlic) as in the CDWR crop map. The USDA and Congalton et al (1998) results indicate that incorporating Landsat images that span the entire year will allow for classification of a wide variety of crops and enable the identification of fields where multiple crops are grown.

Obtaining ground reference data, such as the data collected in the Conglaton et al (1998) study, to use in the classification process is very time consuming and in many cases impossible to collect for generating historical maps. California is unique in that CDWR crop maps based on field site visits are available for specific counties, some dating back to 1976 (http://www.water.ca.gov/landwateruse/lusrvymain.cfm; accessed May 12, 2010). These county-level maps provide historical ground reference data useful for performing crop classification across larger geographical areas and other time periods. For example, crop signatures created from the intersection of the CDWR crop map produced for Fresno County and a time series of Landsat images could be used to classify crop fields in other areas within the Landsat footprint (see Figure 1). The extent of Landsat path 42 row 35 intersects five counties in the Central Valley (Fresno, Kern, Kings, Madera, and Tulare). CDWR maps were available for twelve of the 17 years spanning from 1990 to 2006. As noted above, there are limitations that need to be considered when using the CDWR maps and ground reference samples must be carefully inspected prior to use. Further research is needed to determine the spatial and temporal extent that crop signatures derived from one county can be extended to other areas in the Landsat image and to other years. The next phase of our study is currently focused on this research.

### Limitations of our study

We were unable to study several individual crop types such as melons, squash, cucumbers, onions, and garlic because the CDWR ground reference map we used grouped melons, squash, and cucumbers into one class and onions and garlic into one class. The USDA 2007 crop map classifies these crops separately but was not available at the time of our study. The PUR records list pesticide use on these crops individually and further break down melon (watermelon, cantaloupe) and squash (squash, summer squash, winter squash). It will be important to classify individual crop species to the extent possible to differentiate specific pesticide use. In some cases crop groups with similar pesticide use and timing may be a sufficient degree of identification.

The choice of Landsat spectral bands, or indices derived from the spectral bands, should be carefully considered. We used the NDVI, one of the most widely used indices, to measure vegetation changes over the growing season. There are many other vegetation indices that could be evaluated to determine the optimum indices or spectral bands to measure soil and vegetation properties [32]. Indices derived from the middle infrared spectral bands have been found more useful in some cases than the NDVI measure, which is derived from only the red visible and near infrared bands [33-35]. For example, a recent study by Serra and Pons (2008) evaluated field dynamics using 36 Landsat images for four agricultural classes spanning the years 2002 through 2005 [35]. They found both the NDVI and the Tasseled Cap Wetness (derived from a combination of visible and infrared spectral bands) indices were useful for characterizing crop greenness and moisture conditions over the year.

Our study was limited to the characterization of crop phenology for only one year. Further research is needed to evaluate year-to-year variability of these characteristics. We do not anticipate substantial changes in crop phenological patterns in Fresno County because almost all crops are irrigated (97.4% of harvested cropland was irrigated, 2002 Census of Agriculture [16]). Irrigated crops may not be affected as severely by short-term climate fluctuations as compared to non-irrigated crops grown in other areas of California.

## Conclusions

The recent U.S. Geological Survey policy of offering Landsat data at no cost opens up many new opportunities to explore relationships between environment and health. We found the combination of Landsat 5 and 7 image data could improve pesticide exposure assessment in this region by providing information on crop field conditions at or near the time when pesticides are applied for most of the year. Landsat time-series data were useful for identifying idle, single-, and multi-cropped fields. Landsat data will be limited during the winter months due to cloud cover, and for years prior to the Landsat 7 launch (1999) when only one satellite was operational at any given time. We suggest additional research to determine the feasibility of integrating CDWR crop maps and Landsat data to derive crop maps in locations and time periods where maps are not available, which will enable substantial improvements to chemical exposure estimation.

## Competing interests

The authors declare that they have no competing interests.

## Authors' contributions

SKM obtained the Landsat image data, performed the analysis, and drafted the manuscript. MA produced the study area figure, assisted in drafting the manuscript, and interpretation of the CDWR data. JRN helped develop the study concept and assisted in drafting the manuscript. All authors read and approved the final manuscript.
